# No evidence for a role of the serotonin 4 receptor in five-factor personality traits: A positron emission tomography brain study

**DOI:** 10.1371/journal.pone.0184403

**Published:** 2017-09-07

**Authors:** Dea Siggaard Stenbæk, Vibeke Høyrup Dam, Patrick MacDonald Fisher, Nanna Hansen, Liv Vadskjær Hjordt, Vibe Gedsoe Frokjaer

**Affiliations:** 1 Neurobiology Research Unit, the Neuroscience Centre, Copenhagen University Hospital Rigshospitalet, Copenhagen, Denmark; 2 Center for Integrated Molecular Brain Imaging and Center for Experimental Medicine Neuropharmacology, Copenhagen University Hospital Rigshospitalet, Copenhagen, Denmark; 3 Faculty of Health and Medical Sciences, University of Copenhagen, Copenhagen, Denmark; Ariel University, ISRAEL

## Abstract

Serotonin (5-HT) brain architecture appears to be implicated in normal personality traits as supported by genetic associations and studies using molecular brain imaging. However, so far, no studies have addressed potential contributions to variation in normal personality traits from *in vivo* serotonin 4 receptor (5-HT_4_R) brain availability, which has recently become possible to image with Positron Emission Tomography (PET). This is particularly relevant since availability of 5-HT_4_R has been shown to adapt to synaptic levels of 5-HT and thus offers information about serotonergic tone in the healthy brain. In 69 healthy participants (18 females), the associations between personality traits assessed with the five-factor NEO Personality Inventory-Revised (NEO PI-R) and regional cerebral 5-HT_4_R binding in neocortex, amygdala, hippocampus, and anterior cingulate cortex (ACC) were investigated using linear regression models. The associations between each of the five personality traits and a latent variable construct of global 5-HT_4_R levels were also evaluated using latent variable structural equation models. We found no significant associations between the five NEO personality traits and regional 5-HT_4_R binding (all *p*-values > .17) or the latent construct of global 5-HT_4_R levels (all *p*-values > .37). Our findings indicate that NEO personality traits and 5-HT_4_R are not related in healthy participants. Under the assumption that global 5-HT_4_R levels index 5-HT tone, our data also suggest that 5-HT tone per se is not directly implicated in normal personality traits.

## Introduction

Serotonin (5-HT) is a neurotransmitter involved in multiple normal human behaviors, including sleep, appetite, stress regulation, memory, and emotion processing [1] and in a wide range of neuropsychiatric disorders [2–4]. In healthy humans, 5-HT signaling is proposed to play an important role in personality function although the mechanisms involved are far from clearly understood [5, 6]. Some support for an association between the 5-HT system and personality traits is provided by studies of gene variants of the serotonin transporter (5-HTT) [7, 8] and the rate limiting enzyme in serotonin synthesis (TPH2) [9], however, not all genetic studies have found evidence of an association [10–12]. The five-factor model of personality comprises the personality traits Neuroticism (N), Extraversion (E), Openness (O), Agreeableness (A), and Conscientiousness (C). This model has gained wide acceptance due to converging evidence of its stability, heritability, consensual validation, cross-cultural invariance, and predictive utility [13]. Importantly, scores on certain five-factor traits, e.g., high trait N and low trait C, have been associated with risk for psychopathology [14–16], which may be related to dysfunction of the 5-HT system [17]. Thus, mapping serotonergic underpinnings of personality traits in healthy humans may provide critical insights into the molecular basis of vulnerability for psychopathology.

*In vivo* Positron Emission Tomography (PET) techniques have been used to investigate brain 5-HT markers in relation to normal personality traits including the 5-HT_1A_ [18–22] and 5-HT_2A_ [23–27] receptors as well as the 5-HT transporter (5-HTT) [28–32]. However, methodological issues such as small sample sizes and the use of different psychometric tools to assess personality traits make any overall conclusions difficult. Recently, it has become possible to image the postsynaptic 5-HT_4_ receptor (5-HT_4_R) in humans using the PET radioligand [^11^C]SB207145 [33]. From animal studies there is evidence that 5-HT_4_R binding potential (BP_ND_) is inversely related to 5-HT tonus [34, 35], which has been supported by subsequent genetic [36] and pharmacological intervention [37] studies in humans. Furthermore, we have previously shown that in healthy participants, 5-HT_4_R BP_ND_ is associated with episodic memory [38, 39], familial risk for depression [40], threat-related reactivity of amygdala [41], which is a key brain structure underlying personality trait N [42], as well as trait aggression in healthy males [43]. Together these findings suggest that the 5-HT_4_R may be linked to stable personality characteristics which warrants further investigation.

Thus, we here for the first time evaluate the association between 5-HT_4_R BP_ND_ as imaged with [^11^C]SB207145 PET and five-factor personality traits measured with the NEO Personality Inventory-Revised (NEO PI-R) in healthy participants and hypothesize to detect an association.

## Materials and methods

### Participants

Data from 69 healthy participants (18 females) were available for the study from a large multimodality database established by the Lundbeck Foundation Center for Integrated Molecular Brain Imaging (Cimbi) [44]. Exclusion criteria for the study were history of significant medical disorders, current or previous psychiatric disorders, head trauma, drug and alcohol abuse, current or former use of psychoactive drugs, regular use of medication, non-fluency in Danish, severe somatic illness, pregnancy or breastfeeding. Somatic and psychiatric screening was performed by a trained clinician and included a neurological examination to exclude anomalies. On scan days, participants were asked about use of regular and p.r.n. medication and in some cases (depending on the study design) a urine test was conducted to screen for drug use. Educational scores were rated on a 5-point Likert scale from 1 (no vocational degree) to 5 (> 4 years of academic education including a prior high school degree). Genotype information was available for all but three participants and included BDNF val66met and 5-HTTLPR polymorphisms known to predict 5-HT_4_R [36]. All studies contributing to this work complied with the Helsinki Declaration. Written informed consent was obtained from all participants and the studies were registered and approved by the Copenhagen municipality and the Capital Region Ethics Committee (KF01-2006-20, KF0-274821, H-1-2010-085, and KF-2007-0028,). The data in the current study was collected over a period of 7 years (March 2007 to April 2013) and subsets of the included participants have been part of earlier publications on the 5-HT_4_R [33, 36–41, 43, 45, 46].

### Measures

#### The NEO Personality Inventory-Revised

The Danish version of the NEO PI-R was used to assess personality; this version has previously been normed in a sample of 600 individuals [47]. The NEO PI-R is a self-report questionnaire comprising 240 items which measures five traits of personality: N, E, O, A, and C, where each trait consists of six facets [13]. The participants rated each item on a 5-point Likert scale from 0 (strongly disagree) to 4 (strongly agree). The scores of the items loading on each personality trait were summed to a total raw score, which was used in the analyses. Internal consistency (measured with Cronbach’s alpha, α) for the NEO PI-R traits was high: N, α = .94; E, α = .90; O, α = .87; A, α = .90; and C, α = .92.

#### PET and magnetic resonance imaging (MRI)

The synthesis of [^11^C]SB207145 and quantification of 5-HT_4_R brain BP_ND_ was performed according to previously described procedures [33]. Radiochemical purity of the [^11^C]SB207145 tracer ranged from 95–100%. Two different scanners were used for the PET scans: 1) A High Resolution Research Tomograph (HRRT) with an estimated in-plane resolution of 2 mm [48], and 2) an eighteen-ring GE-Advance scanner with an estimated in-plane resolution of 6 mm (GE, Milwaukee, WI). Immediately following the intravenous bolus injection of [^11^C]SB207145, a 120-min dynamic 3D PET scan (6x5s, 10x15s, 4x30 s, 5x120s, 5x300s, and 8x600s) was conducted. The acquired PET data was reconstructed as described by Fisher *et al*. [36].

MRI was obtained using a 3T Siemens Magnetom Trio scanner (Erlangen, Germany). 2D T2-weighted and high-resolution 3D T1-weighted sequences (matrix 256 x 256; 1x1x1mm voxels) were acquired and corrected for non-uniformity and spatial distortions. Using SPM5 (Welcome Department of Cognitive Neurology, London, UK), T1-weighted images were segmented into gray matter, white matter, and cerebrospinal fluid. Each voxel was assigned to the tissue class with the highest probability and this segmentation was applied afterwards for delineation of the region of interest. The T2-weighted images were used for brain masking.

Pvelab was used to automatically outline regions from the structural MRI scan and subsequently determine time-activity curves within each region [49]. The non-displaceable BP_ND_ of [^11^C]SB207145 was modeled with the simplified reference tissue model using PMOD (PMOD Technologies, Zurich, Switzerland) employing cerebellum as a reference region [33], defined as: BP_ND_ = *f*_ND_*B_avail_*(1/*K*_D_), where *f*_ND_ is the free fraction of ligand in the nondisplaceable tissue compartment, *K*_D_ is the dissociation constant, and *B*_avail_ is the concentration of receptors available for binding [50]. Four regions were included in our model: Neocortex, amygdala, hippocampus, and anterior cingulate cortex (ACC), as these regions are critically involved in the brain’s emotional circuitry [51].

#### Genotyping

Genotyping was performed as described previously for the BDNF val66met [36] and 5-HTTLPR polymorphisms [42]. Genomic DNA was extracted from blood using QIAamp DNA Blood Mini Kit (Qiagen, Valencia, CA). DNA quality and concentration were measured using a UV-Vis spectrophotometer (NanoDrop2000, Thermo Scientific). The participants were subsequently grouped in a bivariate fashion into val-val vs. met carriers for the BDNF val66met and LL vs. S-carriers for the 5-HTTLPR polymorphism.

#### Statistics

The associations between the NEO personality traits and natural log-transformed regional 5-HT_4_R BP_ND_ in neocortex, amygdala, hippocampus, and ACC were analyzed using general linear models. Based on previous observations, the models were corrected for age, sex [45, 47], 5-HTTLPR and BDNF val66met polymorphisms, weight adjusted injected mass, and PET scanner type [36].

**Fig 1:**
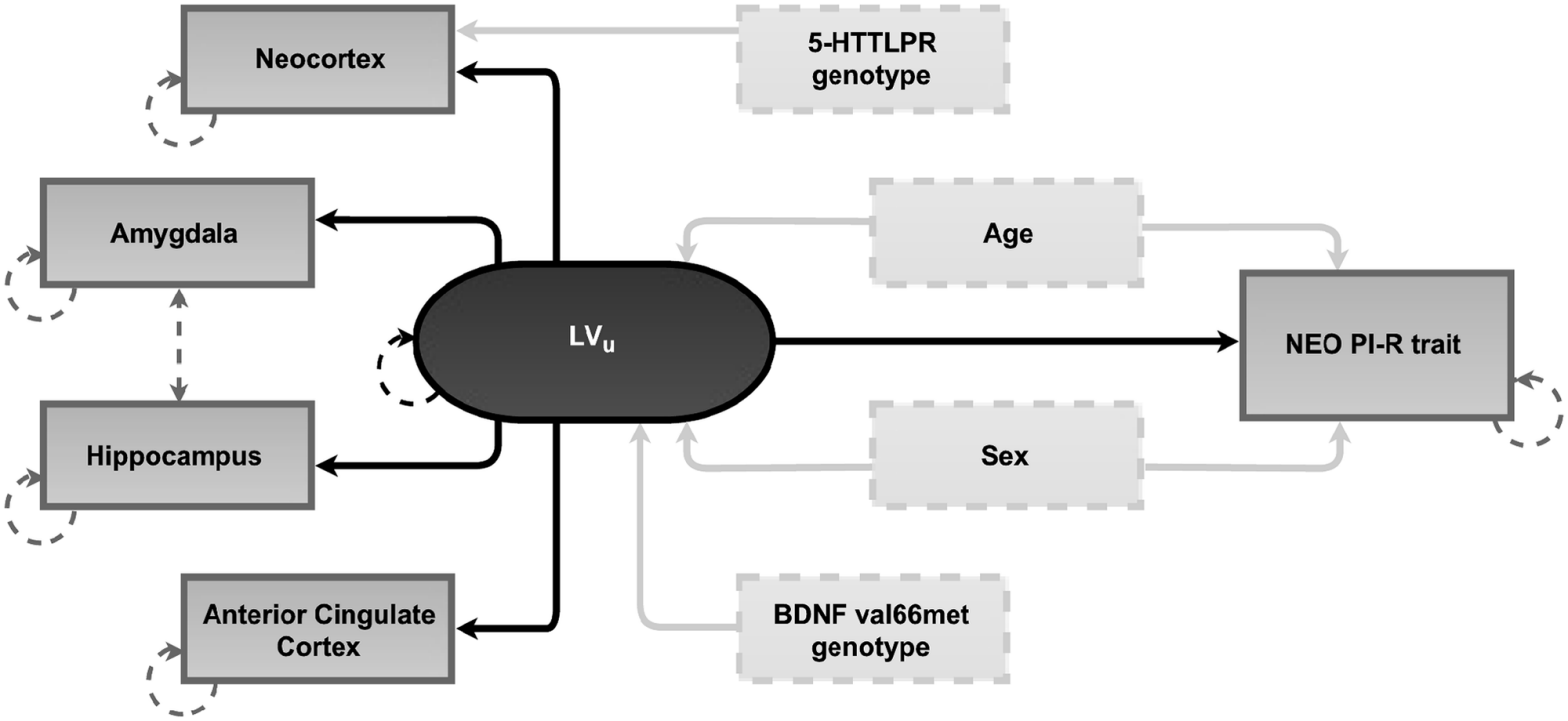
Schematic overview of the latent variable model for the association between cerebral serotonin 4 receptor (5-HT_4_R) binding potential (BP_ND_) and NEO personality traits. A separate model was determined for each of the five NEO personality traits: Neuroticism, Extroversion, Openness, Agreeableness and Conscientiousness. The circle represents the latent 5-HT_4_R variable (LV_u_). Light gray boxes indicate observed predictors and the dark gray boxes, predicted by the latent variable, represent observed log-transformed regional 5-HT_4_R BP_ND_ and NEO personality trait. Age, gender, and BDNF val66met genotype all map directly onto the latent variable (LV_u_). Age and sex also predict the personality trait and 5-HTTLPR genotype predicts neocortex regional BP_ND_ only. Not shown in the model are two observed predictors 1) scanner type (GE-Advance vs. HRRT PET scanner) and 2) weight adjusted injected mass, which map directly onto each regional BP_ND_. The hatched line between amygdala and hippocampus reflects additional shared correlation. Circular hatched lines denote parameters estimated with error.

In line with our previous findings [36], we observed high intercorrelation between 5-HT_4_R BP_ND_ across the regions of interest (*r*-values ranged from .52 to .95, all *p*-values < .001). To best capture a proxy for this global 5-HT_4_R signal and evaluate its associations with each of the five personality traits, we therefore performed a series of secondary latent variable structural equation model analyses (Fig 1).

Latent variable models can be considered an extension of linear mixed models for the analysis of multiple repeated measurements, which in this case allows us to model the shared correlation between different regional brain BP_ND_ as a single variable. A major benefit of using latent variable models, as compared to linear mixed models, is that it allows for some measurements to be more correlated than others (i.e., it can account for the BP_ND_ of a brain region to be differentially correlated to other regions). Furthermore, it substantially reduces the penalty associated with multiple comparisons.

In these analyses, a single latent 5-HT_4_R variable (LV_*u*_) modeled the shared correlation between natural log-transformed regional 5-HT_4_R BP_ND_ in neocortex, amygdala, hippocampus, and ACC. BDNF val66met genotype, weight-adjusted injected mass, age, and sex were included as predictors of the latent 5-HT_4_R variable, 5-HTTLPR genotype was modeled as a predictor of neocortex 5-HT_4_R BP_ND_, and PET scanner type was modeled as predictors of 5-HT_4_R BP_ND_ for each region. Age and sex were also modeled as predictors for NEO personality traits to account for effects independent of the relation between 5-HT_4_R BP_ND_ and NEO PI-R scores [47]. Model fitting and parameters estimates were obtained using the Lava package in R [52], using neocortex BP_ND_ as a reference scale. Additional shared covariance between the amygdala and hippocampus was supported based on Score tests (false-discovery rate *q* < 0.05), which specify whether an additional path would significantly benefit overall model fit, and thus included for all models. Statistical analyses were carried out in SPSS (v22.0) and R (v3.0.2) (R Core Team 2013) with *p*-values < .05 considered statistically significant.

## Results

### Descriptive data

Table 1 shows descriptive data for the 69 healthy participants. Age ranged from 20–86 years (33 ± 16, mean ± SD). The reported scores on the five NEO personality traits did not differ significantly from the Danish norm group [47] on trait N and E (*p* > .22), but our sample showed a significantly higher score on trait O (*p* < .001) and significantly lower scores on trait A (*p* < .01) and C (*p* < .01).

**Table 1:** Descriptive data.

Descriptive data (N = 69)	
Age in years	32.9 ± 15.8 (20–86)
Body Mass Index	23.6 ± 2.6 (19.1–31.3)
Sex (female/male)	18/51
Tobacco use (smokers/non-smokers)	11/57^a^
5-HTTLPR genotype (LL/S-carriers)	28/40^b^
BDNF val66met gentotype (val-val/met-carriers)	30/36^a^
Scanner type (GE-Advanced/HRRT)	20/49
SB specific activity (GBq/mikromol)	126.2 [61.9–211.9]
Neocortex 5-HT_4_ BP_ND_	.58 ± .17 (.24–.88)
Amygdala 5-HT_4_ BP_ND_	.85 ± .18 (.53–1.3)
Hippocampus 5-HT_4_ BP_ND_	.96 ± .18 (.48–1.4)
Anterior Cingulate Cortex 5-HT_4_ BP_ND_	.70 ± .18 (.29–1.1)
Neuroticism	74.0 ± 21.5 (34–139)
Extraversion	116.2 ± 18.9 (53–148)
Openness	115.6 ± 16.5 (73–152)
Agreeableness	119.1 ± 17.0 (47–152)
Conscientiousness	113.3 ± 20.0 (72–150)

Descriptive data for the 69 participants with means ± standard deviation and range in brackets, ratios, or median and interquartile ranges in square brackets. The table shows age, body mass index, sex, tobacco use, 5-HTTLPR LL- and S-carrier ratio, BDNF val66met val/val and met carrier ratio. Scanner type (GE-Advanced vs. HRRT PET scanner), SB specific activity and 5-HT_4_ receptor binding potential (BP_ND_) for neocortex, amygdala, hippocampus, and anterior cingulate cortex are also shown as well as raw scores of the five personality traits: Neuroticism, Extraversion, Openness, Agreeableness, and Conscientiousness measured with the Danish version of the Revised NEO Personality Inventory.

^a^ Data is missing for one participant.

^b^ Data is missing for three participants.

### 5-HT_4_R binding and personality

Consistent with our previous observations [36], the data supported a latent variable model structure, as indicated by a high intercorrelation in 5-HT_4_R BP_ND_ between regions (LV_*u*_: all region BP_ND_ factor loadings across all models, *p* < 1 x 10^−7^). The models for each NEO personality trait all showed good model fit (all *X*^*2*^ < 25.2, df = 20, *p* > .19). The results of the regional analyses are presented in Table 2.

**Table 2:** NEO personality and regional 5-HT_4_R binding potential.

	Neocortex				Amygdala				Hippocampus				Anterior Cingulate Cortex			
	*β*	*p*	95% CI	*r*^2^	*β*	*p*	95% CI	*r*^2^	*β*	*p*	95% CI	*r*^2^	*β*	*p*	95% CI	*r*^2^
Neuroticism	-6.5	.79	[-55.8–42.8]	.07	0.43	.98	[-32.0–31.9]	.06	-9.6	.68	[-55.9–36.7]	.07	-14.1	.52	[-57.6–29.4]	.07
Extraversion	26.3	.22	[-16.3–68.8]	.08	9.5	.49	[-17.8–36.9]	.06	27.8	.17	[-12.1–67.6]	.09	8.8	.64	[-29.2–46.9]	.06
Openness	-12.7	.48	[-48.2–22.8]	.14	0.54	.96	[-22.2–23.3]	.13	3.0	.86	[-30.5–36.5]	.13	-11.8	.46	[-43.2–19.6]	.14
Agreeableness	6.3	.74	[-31.9–44.4]	.09	-1.6	.90	[-25.9–22.7]	.08	21.4	.23	[-14.1–56.8]	.11	18.6	.27	[-14.8–52.0]	.11
Conscientiousness	3.8	.86	[-38.8–46.5]	.18	-2.1	.88	[-29.3–25.1]	.18	10.3	.61	[-29.7–50.3]	.18	12.3	.51	[-25.2–49.9]	.18

Associations between NEO personality traits and regional log-transformed 5-HT_4_R binding potential. *β*, uncorrected *p*-values, 95% confidence interval (CI 95%), and *r*^2^-values are presented. All models were corrected for age, sex, BDNF val66met and 5-HTTLPR genotype, scanner type (Advance vs. HRRT PET scanner), and weight-adjusted injected mass.

Neither regional 5-HT_4_R BP_ND_ in the neocortex, amygdala, hippocampus or ACC nor the latent 5-HT_4_R level (LV_*u*_) showed significant associations with any of the five NEO personality traits (all *p*-values > .17). Neocortex was assigned as the reference region in the latent variable model. Thus, the LV_u_ is interpreted in terms of neocortex BP_ND_, i.e., the presented estimates can be read as changes in personality trait score per unit change in neocortex BP_ND_. The results from the latent variable models are presented in Table 3.

**Table 3:** Latent variable model path analyses.

Path analyses	*β*	SE	*p*	95% CI	*r*^2^
Neuroticism ← U	-1.0	17.7	.95	[-35.8–33.8]	< .001
Extraversion ← U	20.5	16.1	.20	[-11.1–52.1]	.05
Openness ← U	-6.4	13.7	.64	[-33.4–20.5]	.006
Agreeableness ← U	3.2	11.8	.79	[-19.9–26.3]	.001
Conscientiousness ← U	-1.5	15.4	.92	[-31.7–28.7]	< .001

Model path analysis values for each of the five latent variable models comparing underlying cerebral 5-HT_4_R binding potential to the NEO personality traits. *β*, standard error (SE), *p*-values, 95% confidence interval (95% CI), and *r*^2^-values are presented for each of the five model runs. *P*-values denote the significance of each model path and are uncorrected for multiple comparisons.

## Discussion

This is the first study to investigate the association between *in vivo* 5-HT_4_R BP_ND_ and NEO personality traits in humans. Contrary to our expectations, neither regional 5-HT_4_R BP_ND_ in the neocortex, amygdala, hippocampus, and ACC nor the latent 5-HT_4_R level were significantly associated with any of the five NEO personality traits in healthy participants.

Although we observed no association between 5-HT_4_R BP_ND_ and normal personality traits, *in vivo* PET studies with other brain imaging markers of the 5-HT system have been applied to study this relation. Based on these studies, personality trait N appears to be most consistently linked to features of the 5-HT system: Hirvonen *et al*. [19] reported an inverse association between trait N and global 5-HT_1A_ receptor (5-HT_1A_R) BP_ND_ in healthy participants (n = 34), however, two other studies did not observe this relation (Karlsson *et al*. [20] (n = 20), Rabiner *et al*. [21] (n = 44/49)). Frokjaer *et al*. found that trait N was positively associated with frontolimbic 5-HT_2A_ receptor (5-HT_2A_R) BP_ND_ in 83 healthy participants [23] and in 21 healthy participants with a familial risk of mood disorder [24]. Furthermore, Takano *et al*. [30] reported a positive correlation between trait N and 5-HTT BP_ND_ in thalamus (n = 31). Together, these findings support a coupling between certain features of the 5-HT system and personality traits associated with vulnerability for psychiatric disorders [17]. Our data intriguingly suggest that these earlier observed associations between trait N and postsynaptic features of the 5-HT system and 5-HTT are uncoupled from an association with 5-HT_4_R, which presumably captures variations in synaptic 5-HT levels.

High levels of trait N have previously been found to correlate with increased levels of aggression [53]. Therefore, the findings that trait N is coupled with certain features of the 5-HT system correspond with the findings from da Cunha-Bang *et al*. [43] who reported a significant positive association between 5-HT_4_R BP_ND_ and trait aggression in males in a subset of the participants included in the present study. However, the fact that we did not observe any association between 5-HT_4_R BP_ND_ and NEO personality traits suggests that the variance in trait aggression captured by 5-HT_4_R BP_ND_ may be different from the variance shared between NEO personality traits and trait aggression. When we tested the overlap between trait aggression and trait N, we found a shared variance of approximately 25% in our population, supporting that the two could very well be differentially associated with features within the 5-HT system.

Other normal personality traits have shown sporadic associations with 5-HT neurotransmission: Gerretsen *et al*. [25] (n = 24) reported a negative association between 5-HT_2A_R BP_ND_ and trait Reward Dependence measured with the Temperament and Character Inventory (TCI) [54], while Soloff *et al*. [27] found a positive correlation between 5-HT_2A_R BP_ND_ and trait Persistence measured with TCI (n = 21). Lastly, Kalbitzer *et al*. [28] found that 5-HTT BP_ND_ was negatively related to trait O (n = 50), while Tuominen *et al*. [32] observed a positive association between 5-HTT BP_ND_ and trait Self-directedness measured with TCI (n = 22). Notably, only Kalbitzer *et al*. [28] used NEO PI-R to index normal personality traits while the remaining studies used TCI which makes any direct comparisons to our results difficult. In addition, only a few studies used correction for multiple comparisons or compared the personality trait scores of the included participants to established norm data. This may potentially have weakened the strength and generalizability of the reported findings. In reviewing these results, it appears that no clear trend exists with regard to associations between other (i.e., non-trait N) personality traits and 5-HT neurotransmission. Note, only studies with sample sizes ≥ 20 healthy participants are included above. A full review of the existing literature can be found in supporting information (S1 Table).

Given that 5-HT_4_R BP_ND_ has previously been linked to fluctuations in 5-HT tone within the brain [37], our negative finding implies that normal personality traits are not directly related to synaptic 5-HT in healthy participants. Intriguingly, other features of the 5-HT brain architecture which are strongly genetically determined, such as the 5-HT_2A_R [55], or partly represent serotonergic wiring, namely 5-HT neurons and axons such as the 5-HTT [56], do appear to correlate with normal personality traits in healthy participants, especially trait N, as reviewed above. We speculate that the wiring of the 5-HT system predominantly established early in brain development [57, 58], rather than dynamic variations in 5-HT tone, is related to long-term stable personality constructs. This also aligns with the notion that NEO personality traits represent stable psychological constructs, which are established early in brain- and psychological development and maturation [13].

### Methodological considerations

While this is the second largest PET study to examine the association between imaging markers of 5-HT signaling and normal personality traits, some limitations of the study should be considered. First, given our sample size of 69 participants, we have sufficient statistical power (*β* = .8) to detect medium to large effect sizes of *r*^2^ ≥ .09. Thus, while it is unlikely that we have overlooked any medium to large effects, we cannot exclude the possibility that potential small effects were not detected. However, we argue that the practical relevance of such small effects may be negligible since a change of one standard deviation in BP_ND_ (14.8%) in our sample corresponds to a change in a given NEO personality trait smaller than the population-based SEM reported for this trait, i.e., the amount of inaccuracy or error inherent in the measured trait [47]. Second, we were not able to address the potential moderating role of sex for the investigated association between 5-HT_4_R BP_ND_ and NEO personality traits due to the limited number of women included. Given that da Cunha-Bang *et al*. [43] found a significant effect of sex for the association between trait aggression and 5-HT_4_R BP_ND_, combined with earlier reported sex differences in 5-HT_4_R BP_ND_ [45], this will be relevant to consider in future studies. Third, participants with current and previous psychiatric disorders were excluded from our study population and we may therefore have underestimated a potential link between 5-HT_4_R BP_ND_ and NEO personality traits in more vulnerable populations, e.g., participants recovered from depression and/or with a family history of mood disorders. Fourth, self-report biases are inherent methodological issues with questionnaire data, e.g., censorship, social desirability biases, or systematic manipulation of answers on items [59]. However, studies of the correlation between self-report scores and ratings by spouse on the NEO PI-R supports the reliability of the traits measured [13].

### Conclusion

We observed no association between normal personality traits and 5-HT_4_R BP_ND_ in a large group of healthy participants. Under the assumption that global 5-HT_4_R levels index 5-HT tone, our data suggest that 5-HT tone per se is not directly implicated in stable personality traits in the adult healthy brain.

## Supporting information

S1 Table Review of studies investigating serotonin markers and normal personality. Review of PET studies investigating the association between serotonin (5-HT) markers and normal personality in healthy participants. *Personality*: EPQ = Eysenck Personality Questionnaire, KSP = Karolinska Scales of Personality, NEO PI-R = Revised NEO Personality Inventory, TCI = Temperament and Character Inventory, TPQ = Tridimensional Personality Questionnaire. *Brain*: 5-HT = serotonin, 5-HT_1A_ = serotonin 1A receptor, 5-HT_2A_ = Serotonin 2A receptor, 5-HTT = serotonin transporter, ACC = anterior cingulate cortex, BP_ND_ = Binding potential, DLPC = dorsolateral prefrontal cortex, FC = frontal cortex, LPC = left parietal cortex, lt.MFC = left medial frontal cortex, OC = occipital cortex, OFC = orbito frontal cortex PC = parietal cortex, RN = raphe nuclei.
(DOCX)
