# Supplementary material for: No evidence for a role of the serotonin 4 receptor in five-factor personality traits: A positron emission tomography brain study
Source: PLoS One. 2017 Sep 7;12(9):e0184403. doi: 10.1371/journal.pone.0184403 (PMC5589219; doi:10.1371/journal.pone.0184403)
Supplement: S1 Table — Review of PET studies investigating the association between serotonin (5-HT) markers and normal personality in healthy participants. Personality: EPQ = Eysenck Personality Questionnaire, KSP = Karolinska Scales of Personality, NEO PI-R = Revised NEO Personality Inventory, TCI = Temperament and Character Inventory, TPQ = Tridimensional Personality Questionnaire. Brain: 5-HT = serotonin, 5-HT1A = serotonin 1A receptor, 5-HT2A = Serotonin 2A receptor, 5-HTT = serotonin transporter, ACC = anterior cingulate cortex, BPND = Binding potential, DLPC = dorsolateral prefrontal cortex, FC = frontal cortex, LPC = left parietal cortex, lt.MFC = left medial frontal cortex, OC = occipital cortex, OFC = orbito frontal cortex PC = parietal cortex, RN = raphe nuclei. (DOCX) [file pone.0184403.s001.docx]

**S1 Table: Review of studies investigating 5-HT markers and normal personality in healthy participants**

|  | **Citation** | **Group** | **Sample size** | **PET-tracer** | **Personality** | **Primary Findings** |
| --- | --- | --- | --- | --- | --- | --- |
| 5-HT_1A_ | Tauscher *et al.* 2001 [1] | Healthy participants | n = 19 | [^11^C]WAY100635 | NEO PI-R | Anxiety (Neuroticism subfacet) was inversely correlated to 5-HT_1A_R BP_ND_ in DLPC, ACC, PC & OC |
|  | Rabiner *et al.* 2002 [2] | Healthy participants | n = 44 (TPQ)/ 49 (EPQ) | [^11^C]WAY100635 | EPQ / TPQ | No association between personality and 5-HT_1A_R binding |
|  | Borg *et al.* 2003 [3] | Healthy participants (male) | n = 15 | [^11^C]WAY100635 | TCI | Self-transcendence was inversely correlated to 5-HT_1A_R BP_ND_ in neocortex, hippocampus and RN with Spiritual Acceptance as the driving subfacet |
|  | Karlsson *et al.*  2011 [4] | Major depression & healthy controls | n = 23, patients n = 20, controls | [^11^C]WAY100635 | TCI | No association between personality and 5-HT_1A_R BP_ND_ |
|  | Hirvonen *et al.* 2015 [5] | Healthy participants | n = 34 | [^11^C]WAY100635 | KSP | Neuroticism was inversely correlated to global 5-HT_1A_R BP_ND_ |
| 5-HT_2A_ | Moresco *et al.*  2002 [6] | Healthy participants | n = 11 | [^18^F]FESP | TPQ | Harm Avoidance was inversely correlated to 5-HT_2A_R BP_ND_ in FC & LPC |
|  | Frokjaer *et al.* 2008 [7] | Healthy participants | n = 83 | [^18^F]altanserin | NEO PI-R | Neuroticism was positively correlated to 5-HT_2A_R BP_ND_ in frontolimbic regions with Anxiety and Vulnerability as the driving subfacets |
|  | Frokjaer *et al.* 2010 [8] | Twins with either low or high risk of familial mood disorder | n = 21, low-risk n = 16, high-risk | [^18^F]altanserin | NEO PI-R | Neuroticism was positively correlated to 5-HT_2A_R BP_ND_ in frontolimbic regions in high-risk but not low-risk twins |
|  | Gerretsen *et al.* 2010 [9] | Healthy participants | n = 24 | [^18^F]setoperone | TCI | Reward Dependence was inversely correlated to 5-HT_2A_ BP_ND_ in ACC & OFC |
|  | Soloff  *et al.* 2010 [10] | Healthy participants | n = 21 | [^18^F]altanserin | TCI | Persistence was positively correlated to 5-HT_2A_R BP_ND_ in lt.MTC |
| 5-HTT | Takano *et al.* 2007 [11] | Healthy participants (male) | n = 31 | [^11^C]DASB | NEO PI-R | Neuroticism was positively correlated to 5-HTT BP_ND_ in thalamus with Depression as the driving subfacet |
|  | Reimold *et al.* 2008 [12] | Major depression & healthy controls | n = 10, patients n = 19, controls | [^11^C]DASB | TCI | No association between personality and 5-HTT BP_ND_ in healthy controls |
|  | Kalbitzer *et al.* 2009 [13] | Healthy participants | n = 50 | [^11^C]DASB | NEO PI-R | Openness was inversely correlated to 5-HTT BP_ND_ in midbrain with Values and Action as the driving subfacets |
|  | Tuominen *et al.* 2013 [14] | Healthy participants | n = 22 | [^11^C]MADAM | TCI | Self-directedness was positively correlated to 5-HTT BP_ND_ in dorsal RN |
|  | Kim  *et al.* 2015 [15] | Healthy participants | n = 16 | [^11^C]DASB | TCI | Self-transcendence was inversely correlated to 5-HTT BP_ND_ in caudal RN and subfacet Spiritual Acceptance was inversely correlated to 5-HTT binding in median RN |

**S1 Table:** Review of PET studies investigating the association between serotonin (5-HT) markers and normal personality in healthy participants. *Personality:* EPQ = Eysenck Personality Questionnaire, KSP = Karolinska Scales of Personality, NEO PI-R = Revised NEO Personality Inventory, TCI = Temperament and Character Inventory, TPQ = Tridimensional Personality Questionnaire. *Brain:* 5-HT = serotonin, 5-HT_1A_ = serotonin 1A receptor, 5-HT_2A_ = Serotonin 2A receptor, 5-HTT = serotonin transporter, ACC = anterior cingulate cortex, BP_ND_ = Binding potential, DLPC = dorsolateral prefrontal cortex, FC = frontal cortex, LPC = left parietal cortex, lt.MFC = left medial frontal cortex, OC = occipital cortex, OFC = orbito frontal cortex PC = parietal cortex, RN = raphe nuclei.

**References**

1. Tauscher J, Bagby RM, Javanmard M, Christensen BK, Kasper S, Kapur S. Inverse relationship between serotonin 5-HT 1A receptor binding and anxiety: A [ 11 C]WAY-100635 PET investigation in healthy volunteers. American Journal of Psychiatry. 2001;158(8):1326-8.

2. Rabiner EA, Messa C, Sargent PA, Husted-Kjaer K, Montgomery A, Lawrence AD, et al. A database of [11C]WAY-100635 binding to 5-HT 1A receptors in normal male volunteers: Normative data and relationship to methodological, demographic, physiological, and behavioral variables. NeuroImage. 2002;15(3):620-32.

3. Borg J, Andrée B, Soderstrom H, Farde L. The serotonin system and spiritual experiences. American Journal of Psychiatry. 2003;160(11):1965-9.

4. Karlsson H, Karlsson J, Hirvonen JK, Hietala J, Hirvonen J, Salminen J. No association between serotonin 5-HT 1A receptors and spirituality among patients with major depressive disorders or healthy volunteers. Molecular Psychiatry. 2011;16(3):282-5.

5. Hirvonen J, Tuominen L, Nagren K, Hietala J. Neuroticism and serotonin 5-HT1A receptors in healthy subjects. Psychiatry research. 2015 Oct 30;234(1):1-6. PubMed PMID: 26337006. Epub 2015/09/05. eng.

6. Moresco F, Dieci M, Vita A, Messa C, Gobbo C, Galli L, et al. In vivo serotonin 5HT 2A receptor binding and personality traits in healthy subjects: A positron emission tomography study. Neuroimage. 2002;17(3):1470-8.

7. Frokjaer VG, Mortensen EL, Nielsen FÅ, Haugbol S, Pinborg LH, Adams KH, et al. Frontolimbic serotonin 2A receptor binding in healthy subjects is associated with personality risk factors for affective disorder. Biological psychiatry. 2008;63(6):569-76.

8. Frokjaer V, Vinberg M, Erritzoe D, Baaré W, Holst K, Mortensen E, et al. Familial Risk for Mood Disorder and the Personality Risk Factor, Neuroticism, Interact in Their Association with Frontolimbic Serotonin 2A Receptor Binding. Neuropsychopharmacology. 2010;35(5):1129-37.

9. Gerretsen P, Graff-Guerrero A, Menon M, Pollock B, Kapur S, Vasdev N, et al. Is desire for social relationships mediated by the serotonergic system in the prefrontal cortex? An 18 Fsetoperone PET study. Social Neuroscience. 2010;5(4):375-83.

10. Soloff PH, Price JC, Mason NS, Becker C, Meltzer CC. Gender, personality, and serotonin-2A receptor binding in healthy subjects. Psychiatry Research - Neuroimaging. 2010;181(1):77-84.

11. Takano A, Arakawa R, Hayashi M, Takahashi H, Ito H, Suhara T. Relationship Between Neuroticism Personality Trait and Serotonin Transporter Binding. Biological Psychiatry. 2007;62(6):588-92.

12. Reimold M, Batra A, Knobel A, Smolka M, Zimmer A, Mann K, et al. Anxiety is associated with reduced central serotonin transporter availability in unmedicated patients with unipolar major depression: a [C-11]DASB PET study. Mol Psychiatr. 2008;13(6):606-13.

13. Kalbitzer J, Frokjaer VG, Erritzoe D, Svarer C, Cumming P, Nielsen FÅ, et al. The personality trait openness is related to cerebral 5-HTT levels. Neuroimage. 2009;45(2):280-5.

14. Tuominen L, Hirvonen J, Laine J, Hietala K, Tuominen P, Hirvonen T, et al. Temperament, character and serotonin activity in the human brain: A positron emission tomography study based on a general population cohort. Psychological Medicine. 2013;43(4):881-94.

15. Kim JH, Son YD, Kim JH, Choi EJ, Lee SY, Joo YH, et al. Self-transcendence trait and its relationship with in vivo serotonin transporter availability in brainstem raphe nuclei: An ultra-high resolution PET-MRI study. Brain Res. 2015 Dec 10;1629:63-71. PubMed PMID: 26459992. Epub 2015/10/16. eng.
